# 

**Published:** 1906-08-18

**Authors:** 


### BOOKS RECEIVED.

J. and A. Churchill.

"Essentials of Surface Anatomy." By C. R. Whittaker, L.R.C.S.

"Surgery: Its Theory and Practice." By W. J. Walsham, F.R.C.S. 9th Edition.

J. Bale, Sons and Danielsson, Ltd.

"Animal Parasites of Man." By Dr. Max Braun. 3rd Edition.

Cassell and Co.

"How to Find and Name Wild Flowers." By T. Fox, F.L.S.

Hirschfeld Bros.

"Laputa Revisited." By Gulliver Redivivus.

H. K. Lewis.

"The Climate of Lisbon." By Dr. D. G. Dalgado.

The Royal Dental Hospital of London, Leicester Square, has received £500 from the executors of the will of the late Mrs. Hannah Finnie, with the stipulation that the amount be invested and the income therefrom applied for the objects of the hospital.

**Notice to Advertisers and Subscribers.**

All Advertisements, Orders for Copies of The Hospital, and Business Communications should be addressed to **THE MANAGER (Not to the Editor),** The Hospital, 28 & 29 Southampton Street, Strand London, W.C.

The Cost of Subscription, post free, is as follows:—Per week, 3½d.; per quarter, 3s. 3d.; per half-year, 6s. 6d.; per annum, 13s. Foreign and Colonial:—Per annum, 19s. 6d., payable, in all cases, in advance.

**Publications.**

### NEURASTHENIA, Clinical Lectures on.

By THOMAS D. SAVILL, M.D. Lond. 2nd Edition.

*Physician to the Hospital for Diseases of the Nervous System, &c. &c.*

"Difficult to imagine a better or more lucid introduction to the study of neurasthenia."—JOURNAL OF BAL. & CLIMAT. "Throws out hints which are of considerable practical value."—BRIT. MED. JOURN.

H. J. GLAISHER, 57 WIGMORE STREET, W **5s. net; 5s. 4d. by post.**

### WORKS BY DAVID WALSH, M.D.

**RÖNTGEN RAYS IN MEDICAL WORK.** *Third Edition.* 12s. 6d.
**THE HAIR AND ITS DISEASES.** 2s. 6d. net. A Standard Monograph. Baillière, Tindall, & Cox, London.
**GOLDEN RULES OF SKIN PRACTICE.** 1s. net. *Second Edition.* Wright & Co., Bristol.
**AGE AND OLD AGE: Handbook.** 2s 6d. R. A. Everett & Co., London.

JUST PUBLISHED. Price **2s. 6d.** net.

### CARCINOMA OF THE RECTUM.

By F. SWINFORD EDWARDS, F.R.C.S.,
Surgeon to the West London Hospital; Senior Surgeon to St. Peter's Hospital for Stone, and to St. Mark's Hospital for Fistula, &c.

London: BAILLIÈRE, TINDALL, & COX, 8 Henrietta Street, Covent Garden.

THE MOST EFFECTUAL BANDAGE FOR THE CURE OF ULCERS AND OTHER DISEASES OF THE LEG.

### NOTES ON GENERAL PRACTICE.

By S. M. HEBBLETHWAITE, M.D. Lond.

"Sound common sense."—*Hospital.* "We can heartily recommend."—*B. M. Journal.* "A shrewd practical knowledge of everyday general practice."—*Lancet.* "Well worth perusal."—*Scottish Med. & Surg. Journal.* "A great deal of sound advice."—*Edinburgh Med. Journal.* "Will not fail to pleasantly interest."—*Glasgow Med. Journal.* "Well fulfils his hope that they may be found useful."—*Medical Review.* "A very great deal indeed of excellent clinical information."—*Dublin Journal of Med. Sciences.*

Post free 3/9. THE SCIENTIFIC PRESS, 28 Southampton St., W.C.

NOW READY.—ALL BOOKSELLERS.
Crown 8vo. 320 Pages. Cloth, Price 3s. 6d. Twenty-one Illustrations.

### THE RISE AND PROGRESS OF HYDROPATHY

IN ENGLAND AND SCOTLAND. By RICHARD METCALFE, The Hydro, Richmond, Surrey, Author of "Essays and Notes on Hydro-therapeutics," "Life of Vincent Priessnitz," &c.
London: SIMPKIN, MARSHALL & CO.

### IMPORTANT TO HOSPITAL SECRETARIES.

**JUST PUBLISHED. Price 8s. 6d. net.**

### Surgical Dressings Stock Book.

This type of book is used by some of the leading Institutions in the country, and has been found to be of the greatest value as a ready means of noting the details of Surgical Dressings, &c., and comparing the items from day to day or month to month. By its use extravagance or waste in any ward is easily detected.

*PUBLISHED BY*

**THE SCIENTIFIC PRESS, LTD.,**

28 & 29 Southampton Street, Strand, London, W.C.

